# Influence of visual information on sniffing behavior in a routinely trichromatic primate

**DOI:** 10.1093/beheco/arae055

**Published:** 2024-07-04

**Authors:** Brigitte M Weiß, Anja Widdig

**Affiliations:** Department of Human Behavior, Ecology and Culture, Max-Planck-Institute for Evolutionary Anthropology, Deutscher Platz 6, 04103 Leipzig, Germany; Behavioural Ecology Group, Institute of Biology, University of Leipzig, Talstrasse 33, 04103 Leipzig, Germany; Department of Human Behavior, Ecology and Culture, Max-Planck-Institute for Evolutionary Anthropology, Deutscher Platz 6, 04103 Leipzig, Germany; Behavioural Ecology Group, Institute of Biology, University of Leipzig, Talstrasse 33, 04103 Leipzig, Germany

**Keywords:** Barbary macaques, food assessment, multimodal information, olfaction, vision

## Abstract

Most catarrhine primates are considered to be strongly visually oriented, obtaining information about conspecifics and their environment from a diversity of visual cues. Other sensory modalities may provide information that is redundant and/or complimentary to visual cues. When cues from multiple sensory modalities are available, these may reinforce or suppress each other, as shown in several taxa ranging from insects to humans. Here, we tested how the presence and ambiguity of visual information affect the use of olfactory cues when exploring food and non-food items in semi-free-ranging Barbary macaques at Affenberg Salem, Germany. We presented monkeys with pipes containing food (peanuts, popcorn), non-food (stones, feces), or no items in transparent or opaque containers and assessed whether animals looked, sniffed, and/or grabbed into the pipes depending on the visibility of the contents (experiment 1). Visual information had no robust effect on sniffing probability, but monkeys were more likely to sniff before any other form of inspection if the can was opaque than if it was transparent. Both visual and olfactory information affected, whether or not monkeys attempted to retrieve the items from the pipes, whereby monkeys showed an overall decrease in the propensity to grab after sniffing. Furthermore, we manipulated the visual appearance of familiar food items (popcorn) with food colorant (experiment 2), which resulted in substantially increased olfactory inspections compared to unmanipulated popcorn. Taken together, reliance on the olfactory sense was modulated by the available visual information, emphasizing the interplay between different sensory modalities for obtaining information about the environment.

## Teaser

If taking a good look does not get you further, a sniff might do the job. Barbary macaques usually rely strongly on vision, but if visual information about items is absent or ambiguous, they revert to using olfaction sooner or more often when exploring items. When there is the chance that what you get is either tasty or disgusting, both visual and olfactory cues affect whether or not monkeys grab for the items.

## Introduction

Animals gain information about their physical and social environment using different sensory channels. Information can be conveyed via vision, audition, olfaction, or other sensory modalities either by a single or multiple sensory channels in combination. In fact, sensory information is frequently multimodal (i.e. encompassing more than one modality) in nature, and integrating information from multiple modalities is widespread throughout the animal kingdom (e.g. Rushmore et al. 2012; Higham and Hebets 2013). Well-known examples include aposematically colored insects, which typically also emit an odor or sound when approached by a predator, or courtship signals encompassing combinations of visual, acoustic, tactile, and/or chemical elements in a range of taxa (e.g. insects, fish, birds, see review in Hebets and Papaj 2005). The different modalities may thereby provide redundant or complimentary information simultaneously or sequentially, which may reinforce or, in other ways, interact with each other, and/or act at different spatial or temporal scales (Hebets and Papaj 2005). As a result, multimodal information may be more accurate, salient or diverse, more robust to noise, more memorable, or available across a larger range than information conveyed via a single modality (Hebets and Papaj 2005; Higham and Hebets 2013; Leonard and Masek 2014).

Many multimodal studies focus on relatively easily recordable modalities like vision and audition, but an increasing number of studies show multimodal interactions involving olfaction as well (reviewed in Hebets and Papaj 2005). The interplay of olfaction with other senses has been studied relatively well in insects and insect–plant interactions (reviewed for bees in Leonard and Masek 2014) but rather little in vertebrates (see Melin et al. 2019). Nonetheless, there is evidence that the availability and salience of information in one modality may affect to what extent vertebrates attend to other sensory modalities, including olfaction, in various contexts (e.g. communication in lizard *Liolaemus pacha,*Vicente and Halloy 2017; foraging bats *Artibeus watsoni* and *Vampyressa pusilla*, Korine and Kalko 2005; odor detection in humans, Gottfried and Dolan 2003).

Most primates are strongly visually oriented animals with 3-dimensional vision and trichromatic vision, albeit not in all taxa (Jacobs 2009; Kawamura 2016). More specifically, catarrhine primates (Old World monkeys and apes) and howler monkeys have evolved routine trichromacy, while all platyrrhine species (New World monkeys) except howler monkeys and night monkeys as well as some strepsirrhines show a polymorphism that results in heterozygous females being trichromatic and homozygous females as well as all males being dichromatic (Tan and Li 1999; Kawamura 2016). Nonetheless, primates in all taxa also routinely rely on olfaction in foraging, social, and sexual contexts (e.g. Drea 2015; Vaglio et al. 2016; Jänig et al. 2018; Kücklich et al. 2019). Importantly, the reliance on olfaction has been suggested to be related to visual capabilities, feeding ecology, and the availability of sensory cues. For instance, dichromatic white-faced capuchins (*Cebus imitator*) sniffed fruits more often than trichromatic conspecifics (Melin et al. 2019, but see Hiramatsu et al. 2009). Similarly, blind humans subjectively value the sense of smell more than sighted humans and olfactory bulb size was found to be larger in early blind people, although there is no robust evidence for a behavioral difference in olfactory abilities between blind and sighted people when corrected for publication bias (Sorokowska et al. 2019). In a foraging context, black-handed spider monkeys (Hiramatsu et al. 2009), but not white-faced capuchins (Melin et al. 2019), were found to sniff more at visually less conspicuous fruits. An interplay between different senses in foraging success was shown in nocturnal gray mouse lemurs (*Microcebus murinus*), which were able to detect their insect prey using either visual, acoustic, or olfactory cues in isolation but performed best when cues from all 3 modalities were available together (Piep et al. 2008). Similarly, folivore Coquerel’s sifakas (*Propithecus coquereli*) required visual and olfactory cues together to reliably identify more nutritious food, while generalist ring-tailed lemurs (*Lemur catta*) could use either modality alone, and frugivore ruffed lemurs (*Varecia variegata*) could use olfactory, but not visual, cues alone to select the more nutritious food items (Rushmore et al. 2012). Hence, current evidence points toward olfaction in primate feeding ecology being relevant primarily for food selection and when visual information about food quality is lacking or ambiguous, although the overall evidence is mixed and data are still too scarce for robust conclusions (Hiramatsu et al. 2009; Nevo and Heymann 2015). Furthermore, with the exception of a few studies in humans, data on the interplay between vision and olfaction are lacking for species that are routinely trichromatic.

Barbary macaques (*Macaca sylvanus*) are feeding generalists (Fooden 2007) with trichromatic vision (Niimura et al. 2018). They use olfaction in a range of contexts but direct the majority (~80%) of sniffs at food (Simon et al. 2024). Here, we investigated the interplay between visual information and olfaction in 2 experimental feeding contexts. In experiment 1, we provided Barbary macaques with food or non-food items in either a visible or non-visible condition to assess (1) if visibility affected the propensity to sniff at the setup during any stage of exploring it, (2) if visibility affected at what stage of the exploration monkeys used olfaction, and (3) the interplay between visual condition, olfaction, and the (attempted) retrieval of items from the setup. We expected monkeys to be more likely to sniff, and use sniffing as the first type of inspection if visual information was absent. We further expected that they use olfactory information gained in the non-visible condition to inform subsequent behavior (i.e. attempting to retrieve the content or not). In experiment 2, we manipulated the visual appearance of a familiar food item, i.e. popcorn, with food dye to assess how the salience in visual information affected sniffing behavior. We expected that popcorn with unfamiliar visual appearance (i.e. dyed popcorn) would be sniffed more frequently than naturally colored popcorn.

## Methods

### Study population

The study was conducted at Affenberg Salem, Germany in February and March 2021. Affenberg Salem is home to ~200 Barbary macaques in 3 naturally formed social groups that range freely year-round within a 20-ha forested enclosure. The park is open to visitors from spring to autumn and is an active site of research year-round. Visitors are restricted to a designated path, while researchers have access to the entire park area. As a result, the monkeys are well-habituated to human presence (see de Turckheim and Merz 1984 for details on the park). The monkeys feed on plants and insects found naturally within the park and are supplemented daily with fruit, vegetables, and grain. Water is available ad libitum in ponds and water troughs throughout the park. Individuals are identifiable from natural markings such as facial pigmentation patterns, coat color, and stature as well as an alphanumeric code tattooed on the inner thigh. Individual life histories, group memberships, and maternal relatedness are known from near-daily monitoring by staff.

### Experiment I: transparent vs. opaque pipes

#### Setup

We experimentally presented the monkeys with different food and non-food items in a visible and non-visible condition at 12 sites throughout the park. Hence, experiments took place in a near-natural environment without spatial or temporal restrictions of access. For logistic reasons, we split the experiment into 3 blocks, during each of which 4 sites were active simultaneously. In each block, the 4 active sites were distributed across the home ranges of all 3 groups. Sites were set up in locations routinely used by the monkeys but not directly at the feeding areas.

**Fig. 1.**
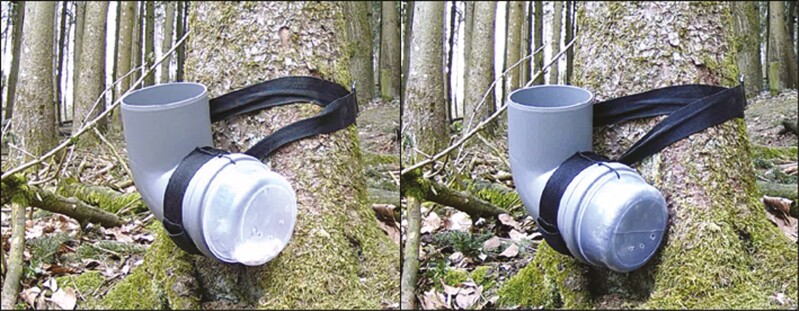
Setup for the pipe experiment with a transparent (left) or an opaque (right) storage can affixed to a drainage pipe. In the example shown, both cans are baited with popcorn, but the popcorn is only visible from the outside when the can is transparent. The setup is open at the top, and storage cans are punctured at the bottom to allow for olfactory investigation from either the top or the can.

At each site, we fixed a gray drainage pipe (11 cm diameter) with a 90° angle to the bottom of a tree. One, open end of the pipe faced upwards, the other to the right (when viewed from the front) as depicted in Fig. 1. The end pointing to the right was closed with a transparent or an opaque food storing can (made of hard plastic, 11 cm diameter) using cable ties. The opaque storing can be identical to the transparent one but coated with a layer of gray duct tape (without appreciable odor to the human observers) on the inside. While we cannot fully exclude that monkeys may have been able to smell the tape inside the can, this should have no effect on whether or not monkeys sniffed, or when monkeys sniffed first (see description of response variables below) because any potential odor of the tape would only have become available after already having sniffed. Due to the angle and dimensions of the pipe, items placed into the can were not visible (as assessed separately by 3 different human experimenters) from the top opening of the pipe, but only through the walls of the can if this was transparent (Fig. 1). All cans were punctured 3 times at the bottom to allow the odors of the content to be perceivable not only from the top opening of the pipe but also when sniffing the bottom of the can (see Fig. 1). Pipes were fixed to trees with black belt straps (38 mm width, polypropylene), with the opening at a height of 30–40 cm. The dimension and height of the setup allowed monkeys to inspect the setup and also to grab into it from the top to retrieve items from the can.

At each site, approaches to and interactions with the setup were continuously monitored with 2 wildlife cameras (Crenova 4K 20 MP) mounted to neighboring trees to cover the setup from 2 different angles. Cameras were set to record a full HD video (avi format with 1,920 × 1,080p resolution) for 1 min without sound whenever detecting movement with their motion detectors (see Supplementary Material S1 for details). Cameras were equipped with a night mode which used infrared light, allowing for 24/7 monitoring.

#### Experimental design

At each site, we first conducted a 2-d habituation phase to familiarize the monkeys with the setup and its location. During the entire habituation phase, a transparent can was attached to the pipe, and the pipe was filled with food items (3 to 6 pieces of peeled peanuts or popcorn) at random intervals 6 to 8 times per day.

The habituation phase was followed by a 7-d test phase. In the test phase, the transparent can was exchanged with an opaque can every few hours and the can was filled with different food or non-food items or was pretended to be filled but left empty (see details below). At each site, the setup was filled 6×/d using the following semi-randomized manner: we divided daylight hours (7 a.m. to 7 p.m.) into six 2 h time slots. In each slot, a given setup was filled once within the first hour of the slot, whereby we randomly determined the start time (0, 15, 30, or 45 min of the first hour) for filling the first setup and the order in which the 4 sites deployed simultaneously were visited. This ensured that the experiment covered the different daytimes evenly without making the visits to the respective setups easily predictable. In this way, there further was at least one hour between consecutive visits to the same site, thereby increasing chances that one or several monkeys passed the setup while filled with a given content.

At each visit, the can was filled with one of 7 possible contents (5 types of contents, 2 of them in 2 different quantities as described below). To provide an incentive for exploring the setup with different senses rather than grabbing inside by default, we did not only fill the setup with food items but also non-food of similar size to the food items or nothing at all. As food items, we either used pieces of peeled peanuts or popcorn, which were provided in 2 different quantities (3 or 6 pieces) to vary the intensity of their odor. As non-food items, we either used 3 stones or 3 small pieces of Barbary macaque feces. We chose stones as presumably neutral, odorless items (i.e. neither particularly desirable nor undesirable for the monkeys). Stones with an approximate size of the peanut pieces were collected within the park area, washed with a neutral soap and water, and then rinsed with distilled water before use in the experiment. In contrast, feces were used as a potentially aversive item, as contact with feces may increase the risk of contracting infections and is avoided in numerous species (e.g. Case et al. 2020; Lopes et al. 2022). Feces were collected opportunistically whenever individually identified; adult monkeys were seen to defecate. Feces were stored in 30 ml glass vials with screw top sealed with parafilm and were frozen at −20 °C within 2 h after collection. Feces were defrosted ~30 min before use and sized to approximately match the size of the peanut pieces. We used feces from both sexes but from other groups than the group using the area of the experimental site. As the experiment was conducted in a near-natural environment without spatial or temporal restrictions in access to the setup, monkeys could potentially observe the filling of the setup. To better disentangle whether a filling event or the items in the setup affected exploratory behavior, we therefore included a 7th possible content, for which the can was pretended to be filled by reaching into it but was left empty. All items were handled with disposable gloves, and the experimenter wore a medical mouth-nose cover. At each visit, potential remains of the previous filling were removed, the inside of the setup was wiped first with 70% Ethanol (Carl Roth) and then with distilled water to remove residues of the ethanol before being refilled.

Over the course of the 7 test days, each of the 7 possible contents was presented once in the transparent and once in the opaque condition in 3 of the six 2 h time slots: one of the first 2, one of the middle 2, and one of the last 2 slots of the day (i.e. 7 contents × 2 conditions × 3 daytimes = 42 times). The order of these 42 filling events was randomized conditional on the following rules: a maximum of 3 consecutive fillings without edible items and no empty conditions twice in immediate succession at a given site (to keep the incentives up), and at least one but a maximum of 3 changes between transparent and opaque cans per day and site (to balance randomization with feasibility). This randomization procedure was done separately for each site so that the order of conditions and contents was also randomized across sites.

#### Coding

We first screened all recorded videos and discarded the “false alarms,” in which the cameras were triggered by movement in the background or other animals than the monkeys (e.g. mice at night). We kept a total of 6570 videos in which one or more monkeys approached (i.e. interacted with or passed within 1 m of) the setup. This number comprises the videos recording the same approach(es) from both angles by different cameras, whereby we only coded one of the simultaneously recorded videos if all behaviors were discernible from a single angle. It further comprises 181 videos recorded in the early morning hours after the end of the 7 d test phase but before the setup was dismantled, which we kept to ensure that the entire time period a setup was accessible to the monkeys was covered.

From the videos, we scored each individual monkey’s approach. Videos could encompass multiple approaches per video or multiple successive videos per approach, if the monkey remained at the setup for more than 1 min. For each approach, we scored from the video the date and start time as well as whether or not the individual looked, sniffed, or grabbed into the setup (see sample videos in Supplementary Material S2a–c). Looking was scored when the approaching animal oriented its eyes toward or into the opening or can of the setup. This was typically accompanied by a clearly noticeable movement and re-orientation of the head from a distance of less than 50 cm, with the eyes closest to the setup while the snout and nose were pulled back toward the neck. Sniffing was scored when an animal placed its nose within 5 cm of the opening or can, with the snout and nose stretched forward and placed closest to the setup. For both looking and sniffing, we differentiated whether the behavior was directed at the opening of the pipe and/or can. Grabbing was defined as moving the hand (and arm) into the opening of the pipe so that at least the entire hand was inside the pipe. If a grab occurred, we also scored whether the monkey successfully retrieved anything from the setup. We scored any other interactions of the monkeys with the setup (e.g. climbing, tugging, biting into it) as “other,” and further coded the order of the respective behaviors. The manipulation of the setup by the monkeys sometimes led to a slight shift in the pipe’s orientation. As a counterclockwise shift could potentially dislodge items from the can toward the bend, where it might have been visible from the opening irrespective of whether the can was transparent or opaque, we also scored the orientation of the pipe’s opening to statistically take into account the possibility that items may have become visible from the top opening (see Supplementary Material S1 for details). All individual identities were scored from video by an observer with 2 yr experience in identifying the study animals. Behaviors were coded from video by a total of 6 different observers blind to the content of the setup, at least in the opaque condition (see Supplementary Material S1 for details and inter-observer reliability).

We determined information on the condition (transparent or opaque) and content of the setup for each approach at each site. For this purpose, we combined information from the experimental schedule (i.e. which type of can was mounted and what was filled into the setup where and when) with information from the videos. While it was not possible to see the actual content of a setup in the opaque condition, it was typically possible for a human observer to see if/what the monkeys retrieved from a can. We could, therefore, update our recorded information about which items had been filled into the respective can at what time with the observations about what the monkeys retrieved in a given approach. In this manner, it was possible for the human observer to deduce the contents of a setup at the start of each approach even if the contents were not visible from the outside.

By combining information on what was filled into the setup when, what was visible in the can on the video, what monkeys retrieved at a given visit to the setup, and what was left in the setup at the next filling event, we were able to confidently deduce the actual content of the setup at the time of each individual approach.

For 7 of the 12 sites, the coded videos encompassed all monkey approaches to the setup (viewed from one or both angles) over the entire test phase. Hence, this also encompassed all approaches after (other) monkeys had already retrieved the items, which represents additional cases of an empty setup similar to when the setup was only pretended to be filled. At the remaining 5 sites, coding all approaches was not feasible because they were visited too frequently. For these sites, we coded no more than the first 7 approaches after a given filling event and discarded the remaining videos (and thereby those approaches in which the setups were likely no longer baited). Overall, we analyzed 3,590 videos, which corresponded to 1,556 different approaches. All approaches took place during full daylight (98%) or late dawn (2%).

### Statistical analysis

To assess the role of visibility on sniffing behavior as well as the consequences of olfactory inspection for further interactions of monkeys with the experimental setup, we constructed 4 generalized linear mixed models (GLMMs) in R (version 4.2.2, R Core Team 2022) using the function glmer in package lme4 (version 1.1-31, Bates et al. 2015). All 4 models were run with binomial error structure with logit link function. The sample sizes used differed between models for analytical reasons as described below and in Supplementary Material S1. As only food, but none of the other items were supplied in different quantities, and a preliminary analysis suggested no differences in sniffing or grabbing behavior in relation to the quantity of items (Roderer 2021), we did not differentiate between quantities in the main analyses (models 1 to 4) to allow fitting all types of contents (peanuts, popcorn, stones, feces, or nothing) in the same model. Details and results of a model investigating a potential effect of quantity on grabbing behavior when the setup contained peanuts or popcorn are presented in Supplementary Material 1.

Model 1 was formulated to investigate the influence of the visibility condition (i.e. transparent or opaque can) on the propensity to sniff at the setup during any stage of exploring it. This model tested the prediction that visibility affects sniffing behavior. We fitted the model with sniff (yes/no) observed as response variable, using 1,354 approaches which represented 853 cases with and 501 cases without sniffs by a total of 97 different individuals. As fixed effects test predictors, we modeled the visibility condition (transparent vs. opaque can), the actual content of the setup at the time of the approach (i.e. peanuts, popcorn, stones, feces, or nothing), and the interaction between visibility condition and content to account for the possibility that different items were more or less likely to elicit sniffs in the transparent condition. We also fitted interactions between visibility condition and 2 control predictors: cohort (i.e. birth year of approaching individual) to account for age-dependent differences in the use of sensory modalities and the number of days a given site was already active to account for a potential learning effect across time. As fixed effects control predictors, we further included sex (male/female), group (C, F, or H), the experimental block (1–3, fitted as a covariate), the time (in min) since the last filling, the orientation of the pipe’s opening (see Supplementary Material S1) and, to account for potential differences in activity patterns, the hour of the day. We further fitted the identity of the coder, the monkey ID, the date, and the site as random intercepts to account for repeated measures and the random variation imposed by these grouping factors.

Model 2 used only data for the 7 sites for which all approaches had been coded (973 approaches by 80 different individuals of which 609 were with and 364 without sniffs). Coding of all approaches allowed us to also determine the time (min) since the previous approach to the setup by the approaching or other individuals, as well as to number the times the approaching individual had previously been at the setup. The motivation of this model was to take into account some of the individual information monkeys may have had available (from own experience or observing others at the setup) in addition to visibility of the content to guide their (olfactory) exploration of the setup. The model was constructed like model 1, but additionally included as fixed effects predictors the time (min) since approach by another monkey (delta other), time (min) since own previous approach (delta self), and nth approach to the setup as well as the 2-way interactions of these terms with the main test predictor, i.e. visibility condition. For the very first approach of an individual to a setup, we imputed the data by using the average time between approaches as time since own previous approach (delta self).

Model 3 was formulated to investigate whether the visibility condition affected if the monkeys used olfaction at the beginning of the exploration or later. Specifically, we expected the monkeys to sniff first when the content was not visible but later during the exploration if it was visible. Hence, this model used only those approaches in which the monkeys did sniff. As a binary response variable, we fitted whether a sniff occurred first or later in the exploration sequence. Hence, 813 approaches by 71 individuals were used in this analysis. These represented 158 cases in which sniffs were the first behavior and 655 cases with sniffs at later stages of the exploration. Similar to model 1, we fitted the visibility condition, the content of the setup, and their interaction, as well as the interaction between the visibility condition and the control predictor day (i.e. number of days the site had already been active) as fixed effects test predictors. As fixed effects control predictors, we further included sex, cohort, group, block, and the hour of the day. We further fitted the identity of the coder, the monkey ID, the date, and the site as random intercepts.

Model 4 specifically investigated the interplay between visual condition, olfaction, and the (attempted) retrieval of items from the setup. We expected that in the transparent condition, vision will primarily guide the decision as to whether individuals would grab or not, while in the opaque condition, olfaction should do so, and that in either case, the decision would depend on the content. We therefore fitted grab (yes/no) as binary response variable, using the same 1,354 cases as in model 1. These comprised 510 approaches with and 844 without grabs. We fitted the visibility condition, the content of the setup, whether or not monkeys sniffed before a grab (yes/no, see Supplementary Material S1), and their 2-way and 3-way interactions as test predictors. As fixed effects control predictors, we further included sex, cohort, group, block, the day of the experiment, the time since the last filling of the setup, and the hour of the day. We also fitted the interaction between the orientation of the pipe and content to account for the possibility that food items (but not non-food items) having shifted into the bend could elicit more grabs. As in the other models, we further fitted the identity of the coder, the monkey ID, the date, and the site as random intercepts.

### General model procedures

To achieve reliable estimates for fixed effects predictors, we fitted all (model 1) or most (models 2 to 4) theoretically identifiable random slopes of fixed effects within the grouping factors (Schielzeth and Forstmeier 2009; Barr 2013, see Supplementary Materials S1 and S3).

For all models, we z-transformed covariates to a mean of 0 and an SD of 1 prior to fitting the model to facilitate convergence and interpretation of model estimates (Schielzeth 2010). To facilitate comparisons between studies, means and SDs of the untransformed covariates are provided in Supplementary Material S4. We further transformed strongly skewed predictor variables as described in Supplementary Material S1.

We checked for collinearity between predictors and assessed model stability, none of which showed severe issues (see Supplementary Material S1). For inference, we conducted Likelihood Ratio Tests as described in Supplementary Material S1. If a model contained non-significant interactions involving test predictors, we excluded the non-significant interactions from the model to facilitate interpretation of the main terms. For these models, we present the final models (after removal of non-significant interactions) in the main text and full models (including non-significant interactions) in Supplementary Material S5.

### Experiment II: colored popcorn

In the second experiment, conducted after the pipe experiment, we manipulated the visual appearance of a familiar food item, i.e. popcorn. This experiment was conducted with 32 adult focal animals of both sexes (16 males with ages of 7 to 21 years, 16 females with ages of 6 to 21 years) from 2 of the 3 study groups. All focal animals were already habituated to retrieving peanuts from a shallow plastic container in the course of another study.

With each of these individuals, we conducted 2 sessions (with 1 to 3 d between sessions), in which popcorn was offered in a gray plastic container (the socket plug of a drainage pipe with 7.5 cm diameter and 3 cm height) fixed to a horizontal branch or wooden rail with a belt strap. Each session consisted of a warm-up and a test trial. In the warm-up trial, a single piece of un-manipulated, white popcorn was offered in the container. If the animal participated in the warm-up trial, the container was filled with 4 pieces of popcorn simultaneously, 2 of which were white and the other 2 blue. Blue popcorn was obtained by dissolving a drop of viscous food dye (Dr. Oetker) in the same amount of water. The diluted dye was soaked up with a paper towel and softly dabbed over the entire surface of the popcorn, then left to dry. As we were unable to obtain white food coloring of the same brand, the white pieces of popcorn were left unmanipulated. In blind tastings of dyed and undyed popcorn, 3 experimenters (each tasting 3 pieces) were not able to distinguish between dyed and undyed popcorn, but we cannot exclude minor differences in smell or taste resulting from the dying process. To avoid interference by other monkeys, sessions were conducted when the respective focal animal had voluntarily separated from group members and was either out of sight or was at least 3 m away from other monkeys, and all individuals in the vicinity were lower-ranking than the focal animal. All sessions were conducted by the same experimenter and recorded with a handheld video camera (Panasonic HC-V180), with the experimenter commenting the observed behaviors and colors of the retrieved popcorn pieces while recording. All 32 focal animals could be successfully tested in this manner.

From the videos, we scored for each session the date and time, and for each piece of popcorn, the order of retrieval, its color, whether or not it was sniffed at and/or eaten. All videos were coded by the same observer.

### Statistical analysis

We analyzed the effect of popcorn color on sniffing behavior by fitting a GLMM with sniff (yes/no) as the binomial response variable with logit link. The unit of analysis was each single piece of popcorn offered to the 32 focal individuals in the test trials (*N* = 32 individuals × 4 pieces per session × 2 sessions = 256). As a fixed effects test predictor, we fitted the color of the popcorn (white/blue). To account for the possibility that monkeys might get used to the novel coloration over the course of the experiment, we further fitted the interaction between color and the n^th^ piece of popcorn retrieved (continuous variable from 1 to 8). We controlled for sex (male/female), cohort, hour of the day, and group (C or F) by fitting these terms as fixed effects control predictors, and for monkey ID and date by fitting them as random intercepts. We further fitted all identifiable random slopes of fixed effects predictors within ID and date (see Supplementary Material S3), but no random correlations, as this caused non-convergence of the model. Checks of model assumptions and stability as well as determination of *P*-values for the full model and individual predictors were conducted as described for experiment 1 in Supplementary Material S1.

We initially also intended to assess whether sniffing and visual appearance jointly affected whether or not items would be discarded or eaten, but restrictions in the experimental design as well as a very low prevalence of discarded popcorn prevented us from explicitly testing this.

### Ethical note

Experiments were approved by Affenberg Salem. As all procedures were non-invasive and participation was voluntary, we required no further animal ethics approval.

## Results

### Experiment 1: transparent vs. opaque pipes

We first addressed if visibility affected the propensity to sniff at the setup during any stage of exploring. In the 1,399 approaches with interactions by identified individuals, monkeys sniffed in 439 (59.1%) of 743 approaches when the transparent can was attached to the pipe and in 443 (67.5%) of 656 approaches when the can was opaque. However, although the proportion of sniffs in the opaque condition was slightly higher, the visibility condition had no significant impact on the propensity to sniff when the other predictors were controlled for (Model 1 on 1354 approaches by 97 individuals: full-null model comparison, LRT, χ^2^ = 11.747, df = 11, *P* = 0.383, see Table 1). This remained qualitatively the same when accounting for potential additional information on the contents from own or others’ prior approaches to the setup (Model 2 on 973 approaches by 80 individuals: full-null model comparison, LRT, χ2 = 17.807, df = 17, *P* = 0.401, see Table 1). While not addressed by the full-null model comparison, model results were suggestive of a significant effect of certain control predictors, namely cohort, with younger individuals being more likely to sniff than older ones in Model 1 (Wald test, z = 3.032, *P* = 0.002) and an interaction between cohort and visibility condition in Model 2 (Wald test, z = 2.015, *P* = 0.044), although the latter result was not very stable across models with different random level effects included (see details on model stability in the methods).

**Table 1.** Estimates of the GLMMs investigating the propensity to sniff for all experimental sites (Model 1, *N* = 1,354 approaches) and for fully-coded experimental sites only (Model 2, *N* = 973 approaches).

	Model 1		Model 2	
Term	Estimate	SE	Estimate	SE
Intercept	1.137	0.407	1.156	0.577
Content				
(feces)	0.119	0.411	0.000	0.541
(nothing)	−0.301	0.347	−0.417	0.484
(popcorn)	−0.664	0.406	−1.085	0.523
(stones)	−0.384	0.415	−0.580	0.544
Condition (transparent)	−0.485	0.426	−0.572	0.599
delta_other			−0.117	0.125
delta_self			−0.222	0.138
n^th^_approach			−0.054	0.302
Cohort	0.328	0.108	0.287	0.129
Day	0.131	0.164	0.351	0.150
Sex (male)	−0.128	0.179	−0.045	0.200
Group				
(F)	−0.114	0.265	0.185	0.410
(H)	0.164	0.327	0.125	0.578
Block	−0.079	0.102	−0.084	0.140
delta_fill	−0.159	0.124	−0.128	0.121
Orientation	−0.081	0.071	0.008	0.081
Hour of day	−0.102	0.080	−0.165	0.101
content:condition				
(feces:transparent)	−0.138	0.547	−0.108	0.725
(nothing:transparent)	0.148	0.461	0.318	0.621
(popcorn:transparent)	0.691	0.584	0.927	0.836
(stones:transparent)	0.304	0.554	0.190	0.745
condition (transparent):cohort	0.172	0.136	0.351	0.174
condition (transparent):day	−0.131	0.149	−-0.245	0.227
condition (transparent):delta_other			0.271	0.183
condition (transparent):delta_self			0.232	0.195
condition (transparent):n^th^_approach			0.120	0.190

Values in parenthesis indicate levels relative to the respective reference level (content peanuts, condition opaque, sex female, group C). SE = standard error. *P*-values were not shown because full-null model comparisons were non-significant.

**Fig. 2.**
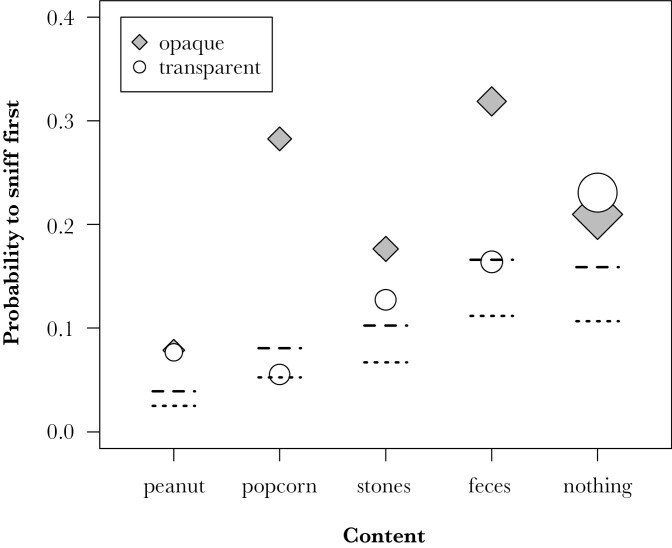
Proportion of approaches with sniffs in which the first interaction with the setup was a sniff depending on content and visibility condition (white symbols: transparent, gray symbols: opaque). Symbols are scaled by sample size (range 38 to 205 approaches with sniffs). Lines depict model estimates (dashed: opaque, dotted: transparent condition) when all other predictors are at their average.

We further assessed if visibility affected at what stage of the exploration monkeys used olfaction. In the 882 approaches during which the monkeys were observed to sniff, sniffing was the very first behavior directed toward the setup in 172 (19.5%) of cases, while the majority of sniffs occurred at later stages of exploring the setup. More specifically, monkeys inspected the setup first by sniffing in 76 (17.3%) of 439 cases in the transparent condition and in 96 (21.7%) of 443 cases in the opaque condition, whereby both content and visibility condition significantly affected the propensity to sniff first (Model 3 on 815 approaches by 71 individuals, full-null model comparison LRT, χ^2^ = 24.782, df = 10, *P* = 0.006, see Table 2). Specifically, monkeys were slightly more likely to sniff first if the can was opaque, irrespective of content, and were more likely to sniff first when non-food items or nothing was in the pipe (Fig. 2).

**Table 2.** Estimates of the GLMM investigating the propensity to sniff first (*N* = 813 approaches with sniffs).

Term	Estimate	SE	χ²	df	*P*
Intercept	−3.061	0.787			
Content			14.061	4	**0.007**
(feces)	1.582	0.536			
(nothing)	1.529	0.507			
(popcorn)	0.766	0.574			
(stones)	1.025	0.566			
Condition (transparent)	−0.458	0.209	4.213	1	**0.040**
Cohort	−0.283	0.185	1.735	1	0.188
Day	0.101	0.141	0.482	1	0.487
Sex (male)	−0.408	0.231	2.933	1	0.087
Group			3.569	2	0.168
(F)	−0.013	0.354			
(H)	0.622	0.409			
Block	−0.209	0.150	1.837	1	0.175
Hour of day	0.084	0.163	0.281	1	0.596

Values in parenthesis indicate levels relative to the respective reference level (content peanuts, condition opaque, sex female, group C). SE = standard error. χ² and *P* values are derived from LRTs to determine the significance of the individual test predictors. Significant effects are marked in bold.

**Fig. 3.**
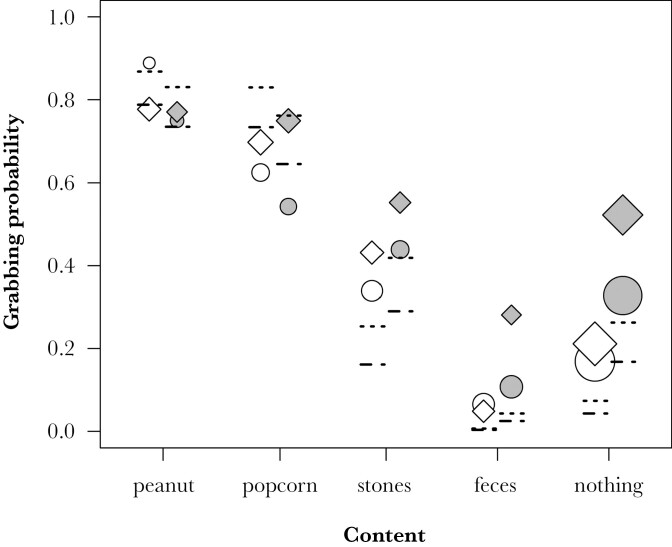
Proportion of grabs into the setup depending on content, visibility condition (white symbols: transparent, gray symbols: opaque), and whether or not monkeys had sniffed first (yes: circles, no: diamonds). Symbols are scaled by sample size (range 18 to 201). Lines depict model estimates (dashed: sniffed first, dotted: no prior sniff) when all other predictors are at their average.

Finally, we investigated the interplay between visual condition, olfaction, and the (attempted) retrieval of items from the setup. In the 1,399 approaches with interactions of identified individuals, the monkeys grabbed into the setup in 516 (36.9%) of cases. Whether or not the monkeys did grab was affected by the content, the visibility condition, and whether or not the monkeys had sniffed at the setup (Model 4 on 1354 approaches by 97 individuals: full-null model comparison, LRT, χ^2^ = 94.424, df = 23, *P* < 0.001). In particular, the effect of the visibility condition depended on the content of the setup (Table 3). Specifically, monkeys showed a similar propensity to grab for food items irrespective of the visibility condition but a reduced propensity to grab for non-food items or into an empty pipe in the transparent versus the opaque condition (Fig. 3). When the monkeys did grab, they actually retrieved peanuts and popcorn in almost all instances of grabbing (peanuts: 95 out of 96 times, popcorn 115 out of 119 times), but only in half of the 80 cases in which they grabbed stones and never in the 24 grabs when feces were inside the can. Sniffing generally decreased the propensity to grab into the setup (Table 3). Although this decrease appeared to be more pronounced in the opaque condition (Fig. 3), the interaction between sniffing and visibility condition or content was not significant. Furthermore, the propensity to grab inside was higher in younger animals and later in the experiment (Table 3). The quantity of the provided food items did not significantly affect grabbing behavior (Supplementary Material S1).

**Table 3.** Estimates of the GLMM investigating the propensity to grab into the setup (*N* = 1,354 approaches).

Term	Estimate	SE	χ²	df	*P*
Intercept	0.522	0.647			
Content					^*^
(feces)	−4.687	0.766			
(nothing)	−2.622	0.521			
(popcorn)	−0.424	0.613			
(stones)	−1.917	0.596			
Condition (transparent)	0.294	0.630			^*^
sniff first (no)	0.568	0.274	4.348	1	**0.037**
Cohort	0.971	0.230	10.366	1	**0.001**
Sex (male)	0.186	0.375	0.248	1	0.619
Group			2.134	2	0.344
(F)	0.492	0.451			
(H)	0.789	0.579			
Day	0.243	0.139	3.125	1	0.077
Block	0.713	0.147	14.216	1	**< 0.001**
delta_fill	−0.163	0.099	2.695	1	0.101
Orientation	0.073	0.142	0.269	1	0.604
Hour of day	−0.152	0.102	2.140	1	0.144
content:condition			15.018	4	**0.005**
(feces:transparent)	−2.173	1.009			
(nothing:transparent)	−1.791	0.688			
(popcorn:transparent)	0.124	0.886			
(stones:transparent)	−1.047	0.781			

Values in parenthesis indicate levels relative to the respective reference level (content peanuts, condition opaque, sniff first yes, sex female, group C). SE = standard error. χ² and P values are derived from LRT to determine the significance of the individual test predictors. Significant effects are marked in bold.

^*^Not presented because terms comprised in significant interaction and thus having a very limited interpretation.

### Experiment 2: colored popcorn

The 32 focal animals retrieved all of the 256 pieces of popcorn and sniffed 58 (22.7%) of them. The color of the popcorn had a clear impact on the probability of sniffing (full-null model comparison, LRT, χ^2^ = 10.77, df = 2, *P* = 0.005). More specifically, blue popcorn was sniffed significantly more often (50 out of 128 times) than white one (8 out of 128 times), irrespective of how many pieces had already been retrieved (see Table 4). Furthermore, females generally sniffed a higher proportion of popcorn than males (Tables 4 and 5). Finally, the sniffing frequency appeared to decrease slightly over the course of the experiment irrespective of color, but this was only a statistical trend and not very robust (see details on model stability in the methods).

**Table 4.** Number (*N*) and fitted values for proportion (prop. fit) of white and blue popcorn pieces sniffed by female and male Barbary macaques.

	White		Blue	
	*N*	prop. fit	*N*	prop. fit
Female	6	0.044	34	0.490
Male	2	0.008	16	0.148

The total number of popcorn pieces per sex and color was 64 (16 individuals × 2 pieces × 2 sessions).

**Table 5.** Estimates of the GLMM investigating the propensity to sniff at popcorn (*N* = 256 pieces of popcorn).

Term	Estimate	SE	χ²	*P*
Intercept	−3.274	0.729		
Color (blue)	3.037	0.675	9.550	**0.002**
Popcorn_nr	−0.829	0.507	3.325	0.068
Sex (male)	−1.709	0.637	7.894	**0.005**
Group (F)	0.334	0.584	0.314	0.576
Cohort	0.043	0.347	0.015	0.902
Hour of day	0.006	0.259	0.001	0.982

Values in parenthesis indicate levels relative to the respective reference level. SE = standard error. χ² and *P* values are derived from LRTs to determine the significance of the individual test predictors. Significant effects are marked in bold.

The monkeys ate the vast majority of popcorn pieces (232) and only discarded 24 of them (23 blue, 1 white). They had sniffed 13 of the discarded blue and the one piece of white discarded popcorn. Due to the low number of discarded pieces (especially white ones) and the lack of a positive control for the dye, we did not assess the interplay between visual and olfactory cues on the decision to eat or discard the popcorn statistically.

## Discussion

This study provides evidence that visual information affects the use of olfaction (i.e. sniffing) and subsequent exploratory behavior of Barbary macaques, but the observed patterns are highly context-dependent. While there was limited evidence that visual information affected the overall propensity to sniff, visual information affected whether monkeys sniffed first or later when exploring the experimental setups. Furthermore, grabbing behavior decreased after sniffing irrespective of the visual context, and sniffing increased when the visual cues of a known food source were experimentally altered.

### Experiment 1: Effects of visual information on olfactory behavior (models 1 to 3)

Sniffing probabilities in experiment 1 were generally rather high and only little affected by the visibility condition: monkeys sniffed in more than half of the approaches even if the content was visible through the transparent can. Notably, sniffing occurred throughout all phases of interacting with the setup. While an initial assessment of the setup using olfaction was slightly more pronounced when cans were opaque than when they were transparent, the majority of sniffs in either visibility condition occurred after the monkeys had actively looked, grabbed or in other ways interacted with the setup. Hence, as suggested for various strepsirrhine and platyrrhine primates (reviewed in Nevo and Heymann 2015) and other mammals (e.g. swamp wallabies, *Wallabia bicolor*, Stutz et al. 2017), sniffing in Barbary macaques may be more relevant *after* an item is located through other sensory modalities (like vision), and thus may function primarily as quality assessment and food selection process rather than in the search for food.

The degree to which vision and olfaction interplay in food search and assessment has been associated with evolutionary adaptations to aspects such as diet or sensory abilities of foragers. In procellariform (“tube-nosed”) sea birds, for instance, differences in light exposure in early development have been related to the degree of dependence on visual and/or olfactory cues during foraging at sea, with burrow-nesting species depending more on olfactory and surface-nesting species more on visual or multimodal cues (Van Buskirk and Nevitt 2008). Similarly, the sensory cues provided by food items and the sensory capabilities of individuals may interact in an intricate manner in primate food selection. In spider monkeys, sniffing decreased from 40% to 45% in fruit species with rather cryptic coloration to 0 for the visually most conspicuous fruit species, irrespective of individual color vision abilities (Hiramatsu et al. 2009). In contrast, Melin et al. (2019) found that dichromatic capuchin monkeys sniffed more at fruits than trichromatic ones irrespective of the conspicuity of the visual cues provided by the fruits, although chromacy-related differences in sniffing rates varied greatly between fruit species and the overall difference was small (8% vs. 7% sniffing in di- vs. trichromatic individuals). Our study animals presumably all had highly similar visual capabilities with the one known exception of a subadult male with one blind eye from birth on. He sniffed in 70% of 37 approaches to the transparent and 73% of 22 approaches to the opaque setup and thus more frequently than the average. However, younger individuals were generally more likely to sniff (and grab into) the setup, and with just one case, we are unable to assess whether the high sniffing rates were related to visual impairment, young age, or just chance. Further studies incorporating a wider range of species are needed to address how aspects such as dynamic vs. stable differences in visual information, diet, and other aspects of species ecology or individual differences in sensory capabilities affect to what extent vision affects olfactory behavior.

### Experiment 1: Effects of sensory information on grabbing for items (model 4)

Monkeys grabbed into the setup in a little more than one third of the approaches. As expected, whether or not monkeys grabbed was modulated by the content and its visibility. They were most likely to grab for peanuts and least for feces in either visibility condition, but the differences in grabbing between contents were less pronounced in the opaque condition. This suggests that the absence of visual information limited the monkeys’ ability to distinguish the contents of the setup, but also that they did have other sensory information than the items’ visibility available to guide the decision as to whether or not to grab inside. Our prediction that olfaction would mediate further exploration of the setup in the absence of visual information, however, was only partly supported. Results indeed indicated a significant effect of prior sniffing on the propensity to grab. Based on grabbing rates (see Fig. 3), this effect appeared to be more pronounced in the opaque condition, particularly when feces, nothing, or popcorn was inside. However, statistically, an interaction between sniffing, visibility condition, and content could not be confirmed; model results rather suggest that monkeys that had sniffed the setup were generally less likely to grab inside irrespective of content or visibility condition. Although we scored well over 1,000 approaches, the study design was complex, and the number of actual approaches to a given content and condition as well as the (sniffing) behavior shown by the monkeys upon approach was not controllable by us, so that for particular combinations of predictors, cases may have still been too rare to detect statistically robust patterns. Another explanation could be that some residues of fecal odor remained in the setup even if other contents were inside, although we took great care to thoroughly clean the setup between fillings. Given the much lower propensity to grab for feces than for other items, this could explain a generally lower propensity to grab after sniffing irrespective of visibility condition and content, but if this was the case, we would have expected a much more pronounced decrease in grabs after sniffing than actually observed. We also consider it unlikely that a potential smell of the duct tape in the opaque condition affected grabbing because sniffing the setup reduced grabbing irrespective of the visibility condition. Notably, even if monkeys had not sniffed, grabbing rates differed between contents in the opaque condition (see Fig. 3), suggesting that the monkeys may have had other sources of information about the contents than anticipated. One possibility is that the odors of the contents may have been perceivable from a greater distance and without requiring the active sniffing movement that we scored, so that we may have underestimated the number of approaches in which olfactory information was available and used by monkeys. Although the majority of studies point toward primates primarily using olfaction for the close-up assessment of food items (Nevo and Heymann 2015), at least some species of strepsirrhines and platyrrhines appear to be able to detect and locate food sources from up to several meters distance using olfactory cues (Bicca-Marques and Garber 2004; Cunningham et al. 2021). Monkeys also sometimes pulled at the setup, which may have dislodged items from the tip of the can into a position visible from the top opening of the pipe. We statistically controlled for this possibility by including the orientation of the pipe into the models but could not unambiguously determine for each approach whether or not monkeys could have seen the contents even in the opaque condition. Sound is unlikely to have provided salient information about the contents (other than maybe that there is something inside). Touch, on the other hand, has been suggested to be an important sense for evaluating food quality in primate feeding ecology (Dominy 2004; Veilleux et al. 2022). For instance, results by Dominy et al. (2016) suggest that the ingestion of green figs, *Ficus sansibarica*, by chimpanzees was elicited by fig toughness rather than their color or size. Along similar lines, the Barbary macaques in the present study may have grabbed inside the setup to gather tactile information about the item. The fact that the monkeys, in case of a grab, almost always retrieved food, but only half of the stones and none of the feces supports the idea that monkeys may have also used tactile cues, but this possibility would need to be assessed systematically in a future study.

Finally, the food items used in the experiment are highly favored by the monkeys, and peanuts, in particular, are not routinely provided by the park. Hence, the incentive of gaining a favorite food item may have been higher than costs for an unsuccessful grab revealing no food or even an item like feces, so that at least some individuals may have pursued the strategy to grab inside as long as there was no obvious cue suggesting feces or other non-food content. Furthermore, monkeys not having encountered feces in the setup in previous approaches may have been more prone to grab inside irrespective of visual or odor cues to the content. However, taking into account the prior setup experience of each individual would have required a much more detailed investigation including coding every single video, which was not feasible in the present study. Taken together, Barbary macaques appeared to depend more on visual than olfactory information to distinguish between items, although question marks remain as to whether or how visual and olfactory information interact to guide exploratory behavior in an experimental feeding context.

### Experiment 2

The second experiment addressed a different aspect of visual information. By presenting blue and white pieces of popcorn, visual information was constantly available but while size and shape were familiar, the color of dyed popcorn was not and thus represented a source of ambiguity. Indeed, monkeys sniffed popcorn in a novel (blue) color considerably more often than the familiar, white popcorn. This matches results in spider monkeys and squirrel monkeys, who used olfaction and other non-visual senses more when presented with novel food (i.e. entirely unfamiliar or with unfamiliar color or scent), while relying primarily on vision for assessing familiar food items (Laska et al. 2007). The unfamiliar visual appearance of the dyed popcorn also appeared to be particularly salient for sniffing behavior in Barbary macaques and elicited stronger changes in olfactory inspections than the presence or absence of visual cues in experiment 1.

Unfamiliar colors have also been shown to affect the acceptance and manipulation of food items (e.g. zebra finches: Kelly and Marples 2004; spider monkeys and squirrel monkeys: Laska et al. 2007). In our popcorn experiment, 23 of the 24 discarded pieces were dyed, but the monkeys consumed such a large proportion of the popcorn pieces (91%) overall that we did not have a sufficient number of discarded pieces to statistically assess how the interplay between visual information and sniffing behavior affected consumption. Furthermore, our inference about the role of visual appearance on food consumption was limited by the fact that we had no white food coloring of the same brand as the blue one available and thus compared dyed with undyed popcorn. Although different experimenters were not able to detect a difference in smell or taste of the dyed and undyed popcorn, we cannot exclude that the monkeys may have been able to perceive a difference. If that was the case though, this information should only have become available after sniffing or tasting the popcorn. Accordingly, a potential difference in the smell or taste of dyed popcorn should have been of little relevance for the question if an unusual color elicits olfactory investigation, but might have had an effect on the consumption of the popcorn. What we may conclude from the limited number of discarded pieces is that the visual appearance mattered, given that the monkeys discarded 10 of the 23 discarded blue pieces without prior sniffing. Nonetheless, the proportion of popcorn discarded after sniffing was twice as high for the dyed compared to the unmanipulated popcorn (26 % vs. 13 %). One possible explanation, therefore, is that the dye changed the smell of the popcorn, but if so, the change was presumably minor given that many more of the dyed pieces were eaten (*N* = 37) than discarded (*N* = 13) after sniffing. Alternatively, the sniffed dyed pieces were not discarded because they smelled differently than the undyed pieces, but because the unfamiliar visual information overrode the familiar olfactory one. Follow-up studies should incorporate different colors as well as combinations with odors (as done in, e.g. Kelly and Marples 2004; Laska et al. 2007) to get a better understanding how unfamiliar visual appearance and olfactory information interact in catarrhine primate food choice.

## Conclusions

This study provides evidence that Barbary macaques used visual and olfactory information to guide exploratory and feeding behavior in experimental feeding contexts. As a highly visually oriented species with acute color vision it comes as no surprise that the presence or absence as well as the salience of visual information clearly affected how monkeys responded to different food and non-food items. Monkeys also routinely used olfaction to inspect items, particularly if visual information was ambiguous (i.e. familiar food in an unfamiliar color), while the presence/absence of visual information had only minor effects on sniffing behavior. Olfactory inspection affected subsequent behavior to some degree, albeit less than vision. As such, this study adds to the increasingly comprehensive evidence that olfaction plays a prominent role in the lives of catarrhine primates, which were historically regarded to rely only little on the sense of smell (Heymann 2006). Importantly, by taking a multimodal approach we were able to show that the availability and salience of information in one sensory modality may affect the use of other sensory modalities, potentially leading to a complex, multisensory interplay affecting behavior. As also pointed out by various authors (e.g. Smith and Evans 2013; Leonard and Masek 2014; Valenta et al. 2015; Wilke et al. 2017; Veilleux et al. 2022), our results highlight the importance of a multimodal perspective, as examining single modalities may be insufficient to explain behavioral decisions. How such multisensory interactions guide behavior in a feeding or other contexts requires further studies and across a wider range of species to understand how (socio)ecological traits shape sensory ecology.

## Supplementary Material

arae055_suppl_Supplementary_Material_S1

arae055_suppl_Supplementary_Material_S2

arae055_suppl_Supplementary_Material_S3

arae055_suppl_Supplementary_Material_S4

arae055_suppl_Supplementary_Material_S5

arae055_suppl_Supplementary_Material_S6

arae055_suppl_Supplementary_Material_S7

## Data Availability

Analyses reported in this article can be reproduced using the data provided by Weiß & Widdig (2024).
